# Biomechanical evaluation of a short-rod technique for lumbar fixation surgery

**DOI:** 10.3389/fbioe.2022.959210

**Published:** 2022-08-11

**Authors:** Ze-Bin Huang, Mao-Dan Nie, Ning-Ze Zhang, Shu Liu, Jia-Bin Yuan, Xu-Miao Lin, Cheng-Kung Cheng, Zhi-Cai Shi, Ning-Fang Mao

**Affiliations:** ^1^ Department of Spine Surgery, First Affiliated Hospital of Naval Medical University, Shanghai, China; ^2^ School of Biomedical Engineering, Shanghai Jiao Tong University, Shanghai, China; ^3^ Key Laboratory of Biomechanics and Mechanobiology, Ministry of Education, Beijing Advanced Innovation Center for Biomedical Engineering, School of Biological Science and Medical Engineering, Beihang University, Beijing, China

**Keywords:** implantation technique, short-rod technique, pedicle screw, screw inclination angle, rod length, biomechanical evaluation

## Abstract

**Objective:** The purpose of this study was to analyze the stability and instrument-related complications associated with fixation of the lumbar spine using the Short-Rod (SR) technique.

**Methods:** Using finite element analysis, this study assessed the stability of a bilateral lumbar fixation system when inserting the pedicle screws at angles of 10°, 15°, and 20° to the endplate in the sagittal plane. Using the most stable construct with a screw angle, the model was then assessed with different rod lengths of 25, 30, 35, and 45 mm. The optimal screw inclination angle and rod length were incorporated into the SR model and compared against traditional parallel screw insertion (pedicle screws in parallel to the endplate, PPS) in terms of the stability and risk of instrument-related complications. The following parameters were evaluated using the validated L4–L5 lumbar finite element model: axial stiffness, range of motion (ROM), stress on the endplate and facet joint, von-Mises stress on the contact surface between the screw and rod (CSSR), and screw displacement.

**Results:** The results showed that the SR model with a 15° screw inclination angle and 35 mm rod length was superior in terms of construct stability and risk of complications. Compared to the PPS model, the SR model had lower stiffness, lower ROM, less screw displacement, and lower stress on the facet cartilage, the CSSR, and screws. However, the SR model also suffered more stress on the endplate in flexion and lateral bending.

**Conclusion:** The SR technique with a 15° screw inclination and 35 mm rod length offers good lumbar stability with a low risk of instrument-related complications.

## Introduction

The function of the spine is to provide flexibility, support the upper body, and protect the spinal cord and nerve roots. Some idiopathic diseases and severe external loads may cause nerve compression and destabilize the spine (Bogduk, 2016). Pedicle screw fixation is considered the gold standard for stabilization of degenerative disk disease (Shih et al., 2012; Chu et al., 2019), and the most common practice is to insert pedicle screws in parallel to the endplate (PPS) and connect them with rods. The effectiveness of the PPS technique in providing primary stability to the lumbar spine is widely recognized (Fang et al., (2022). However, the PPS technique requires extensive tissue dissection to implant the internal fixation device, often resulting in muscle atrophy, back pain, and symptoms of failed back surgery (Fu et al., 2020).

Nonparallel screws can increase the biomechanical stability of the device by converging in the sagittal plane. Farshad et al. (2014) found that sagittal nonparallel screws have at least equal initial fixation strength compared to PPS. Pang et al. (2015) reported that proper angular inclination of the screw can significantly improve the stability of the screw. However, the screws in these studies were oriented in the same oblique orientation. In a clinical study, we noticed that changing the angle for ipsilateral screws to be inserted obliquely with different orientations into the vertebrae in the sagittal plane can tightly connect the tails of the screws so that a shorter rod is required, which allows for implantation through a smaller incision. We named this novel technique as the Short-Rod (SR) technique. Patients with small incisions experience less blood loss during surgery, a quicker return to daily activities, lower cost, and less postoperative chronic back pain and iatrogenic injury (Bresnahan et al., 2017; Momin and Steinmetz, 2020). However, despite the obvious advantages of the SR technique, there is no clear guidance on choosing a suitable screw inclination angle or rod length. In addition, screw fixation alters the biomechanical behavior of spinal structures, and complications associated with this technique have been frequently reported. Previous studies evaluated specific properties of the nonparallel screws, such as the screw pull-out strength (Farshad et al., 2014), but a comprehensive analysis of the biomechanical function and risk of the SR technique has not been considered.

Symptoms of degenerative disc disease are mostly due to instability of the lumbar spine (Bogduk, 2016). Assessing the stability of the lumbar spine, such as ROM and axial stiffness (Christine et al., 2019), is the most important indicator of the effectiveness of surgery. Postoperative complications are a major cause of re-operation and reduced patient quality of life, with endplate fracture, facet joint degeneration, screw loosening, and breakage being the most commonly reported (DieeN et al., 1999; Chang et al., 2017; Nie et al., 2019; Xi et al., 2021). The biomechanical evaluation of screw fixation systems by experimental methods is time-consuming, labor-intensive, and expensive. Finite element model (FEM) analysis can parametrically alter one input factor such as the force or moment and assess its impact on different screw insertion techniques (Kim et al., 2012). It is widely used for the mechanical analysis of lumbar internal fixation systems due to its reproducibility and repeatability (Dreischarf et al., 2014).

This study proposes to use the FEM to investigate the impact on the biomechanics of different screw inclinations and rod lengths using the SR technique, with the aim of defining appropriate parameters for stable fixation. Then, the biomechanical characteristics of the SR technique and PPS technique will be compared under different loading modes. The hypothesis of this study is that the SR technique offers better stability and a lower risk of instrument-related complications than the PPS technique.

## Materials and methods

### Development of an intact lumbar finite element model

**FIGURE 1:**
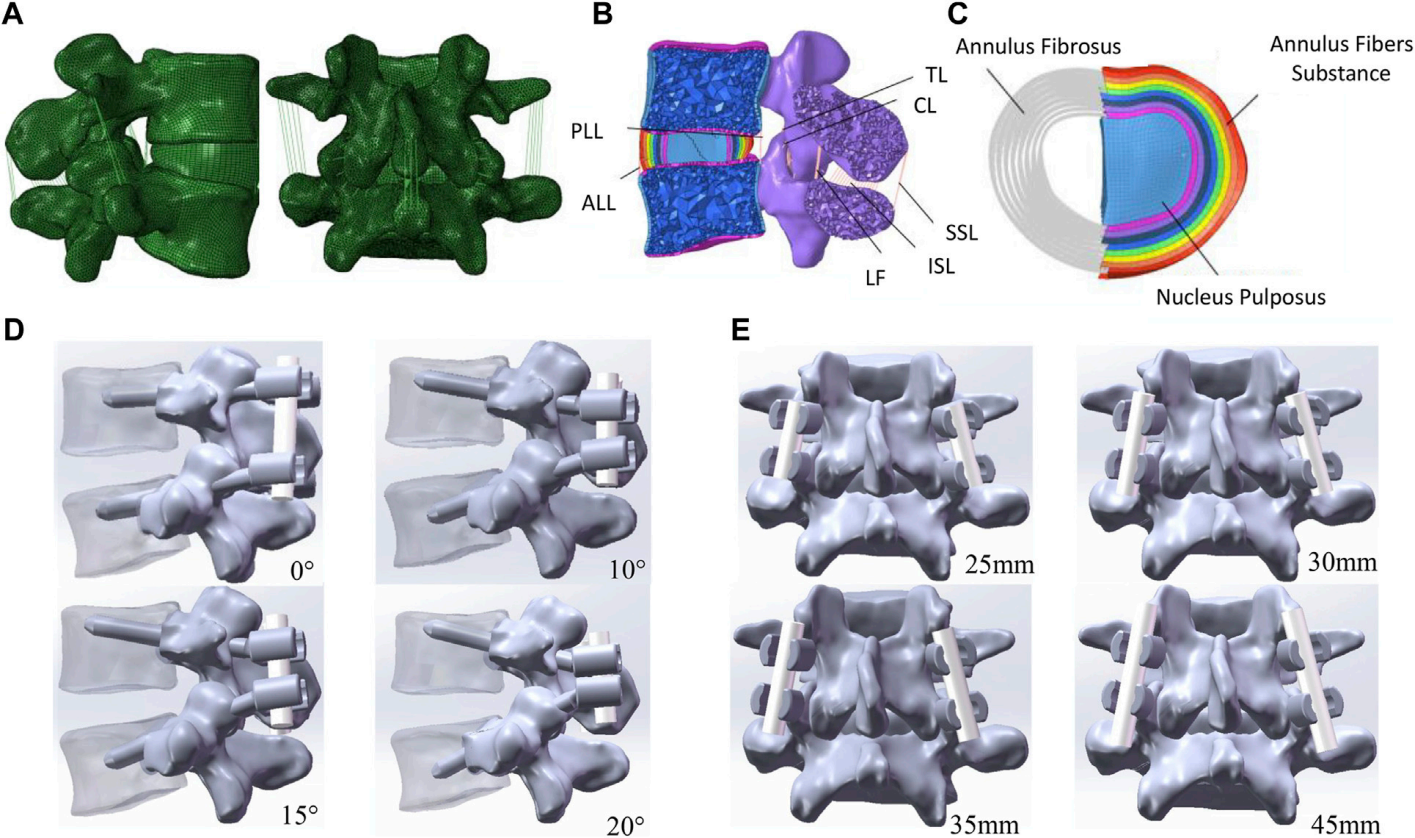
Finite element model of the lumbar spine. **(A)** L4–L5 functional spinal unit. **(B)** Transverse section of L4–L5 spine. **(C)** Intervertebral disc model. **(D)** Instrumented vertebra and pedicle screw model showing different screw angles. **(E)** Instrumented vertebra and pedicle screw model showing different rob lengths.

A 3-dimensional lumbar model consisting of an L4–L5 functional spinal unit was reconstructed from CT scans (SOMATOM Definition AS+, Siemens, Germany) of a 30-year-old healthy female in Mimics 10.01 (Materialise Technologies, Belgium). The CT images were taken with a slice thickness of 0.625 mm. The solid model was built by reverse engineering in Geomagic Studio 10.0 (Geomagic Inc., United States) and Solidworks 2021 (SolidWorks Corp., MA, United States). The solid model mesh was then converted to a meshed model in Hypermesh 17.0 (Altair Engineering Corp., United States). Figure 1 shows the lumbar spine model, which contains the cortical bone, cancellous bone, posterior structure, endplate, nucleus pulposus, annulus fibrosus, and annulus fiber substance. The intervertebral disc height of the model in this study is 9.90 mm, the vertebral body height of L4 is 31.06 mm, and the vertebral body height of L5 is 28.56 mm, all of which are within the range reported in literature (Campbell-Kyureghyan et al., 2005), indicating that the model geometry is universal. Mesh convergence testing of the intact lumbar model was performed in Abaqus 2021 (Dassault Systemes Simulia Inc., France) using stress distribution. The element size was reduced until there was a negligible change in the *in situ* force in the disc. The resulting model consisted of 604,487 elements of 1 mm in size.

The properties of all components in the lumbar spine model are shown in Table 1. Collagen fibers were simulated using tension-only truss elements (T3D2) and embedded in the ground substance of the intervertebral disc with an average angle of ±30° to the endplates to form the annulus fibrosus. The ground substance was composed of eight layers 1.0 mm thick (Zhang et al., 2022). The volume ratio of the annulus fibrosus and nucleus pulposus was 3:7 (Goto et al., 2002). The cortical bone was 1 mm thick, and the endplates on the upper and lower surface of the vertebrae simulated the connection between the vertebrae and the intervertebral disc (Song et al., 2021). The thickness of the cartilage layer of the facet joint was assumed to be 0.2 mm (Kim et al., 2015). All ligaments were modeled using truss elements (T3D2) placed under tension only without compression, which included the anterior longitudinal ligament (ALL), posterior longitudinal ligament (PLL), interspinous ligament (ISL), supraspinous ligament (SSL), ligamentum flavum (LF), ligament intertransversarii (ITL), and capsular ligament (CL). The insertion locations of ligaments were referenced from the anatomical attachment points (Thompson, 2015). Node sharing was set between each ligament and its attachment point to the bone and between each vertebra and disc to increase the efficiency of modeling.

**TABLE 1:** Properties of different components in the lumbar spine model (Zhang et al., 2022).

Components	Young’s modulus (MPa)	Poisson’s ratio	Cross-sectional area (mm)
Cortical bone	12000	0.3	
Cancellous bone	100	0.2	
Posterior bone	3500	0.25	
Endplate	500	0.3	
Nucleus pulposus	1	0.49	
Annulus fibrosus	4.2	0.45	
Annulus fiber layers	360–550	—	0.76
ALL	15.6–20	0.3	63.7
PLL	10–20	0.3	18
LF	13–19.5	0.3	40
CL	7.5–33	0.3	32
ITL	12.0–58.7	0.3	1.8
ISL	8.8–15	0.3	25.2
SSL	9.8–12	0.3	35.1
Screw-rod system	110000	0.3	

### Validation of the intact lumbar finite element model

To validate the FE model, the L4–L5 intervertebral disc was subjected to pure compressive forces of 150, 400, and 1000 N, and the resulting stresses were compared with *in vitro* experimental data (Brinckmann and Grootenboer, 1991; Dreischarf et al., 2014). Considering the limitations on exercise postoperatively, the model was also placed under a 300 N axial (Song et al., 2021) and three different moments (3.0, 7.5, and 10 Nm) to simulate flexion, extension, lateral bending, and axial rotation. The resulting range of motion (ROM) of L4–L5 was compared with *in vivo* experimental results (Goel et al., 1985; Yamamoto et al., 1989; Karahalios et al., 2010). To validate the PPS model, the L4–L5 intervertebral disc was subjected to a 300 N axial and 7.5 Nm, and the resulting von-Mises stresses and ROM were compared with those in previous studies (Liu et al., 2020; Zhang et al., 2021).

### Development of the surgical lumbar finite element model

The L4–L5 spinal segment was secured by bilateral fixation with polyaxial screws (Ø6.0 mm × 45 mm) and rods (Ø3.5). The pedicle screws were titanium alloy (Ti_6_Al_4_V). The coefficient of friction for the contact interfaces between the screws and vertebrae was set as 0.1 (Xu et al., 2013).

In the PPS model, the screws were placed horizontal to the endplate, while in the SR model, the screws were angled at 10° (SR-10°), 15° (SR-15°), and 20° (SR-20°) to the endplate plane (Figure 1D) (Farshad et al., 2014; Pang et al., 2015). The same insertion point was used regardless of screw inclination, and both techniques had the same projection angle on the horizontal plane. All screws were inserted under the instruction of an experienced surgeon.

Few studies discuss the effect of rod length on the screw-rod fixation system, but rod length is related to wound size and should be considered in terms of surgical approach and postoperative recovery. The rod lengths in this study were determined based on the author’s clinical experience; 25 mm (SR-25 mm, tangent to screw head), 30 mm (SR-30 mm, 2.5 mm protrusion from screw head), 35 mm (SR-35 mm, 5 mm protrusion from screw head), and 45 mm (SR-45 mm, 10 mm protrusion from screw head) (Figure 1E).

Spinal stability was analyzed by measuring the range of motion (ROM) and compressive stiffness around the neutral zone of the spinal segment (Chen et al., 1990; Christine et al., 2019). Common instrument-related complications were also assessed, including the risk of endplate fracture, degeneration of facet joints, and screw loosening and breakage (DieeN et al., 1999; Nie et al., 2019; Xi et al., 2021). These risks were assessed using the stress distribution and displacement of the model (Chen et al., 2001).

The lower surface of L5 was fixed in all directions, and a reference point was established on the upper surface of L4 as the point of the force application. The properties were the same as those of the intact lumbar model. The axial compressive stiffness of each model under a 300 N load was determined from the load–displacement curves. Then, a 7.5-Nm moment was added to the surface of the model to simulate flexion, extension, lateral bending, and axial rotation. The ROM, stress on the endplate and facet cartilage, von-Mises stress on the screws and contact surface between the screw and rod (CSSR), and the screw displacement were calculated.

## Results

### Validation of the intact lumbar finite element model and pedicle screws in parallel to the endplate model

**FIGURE 2:**
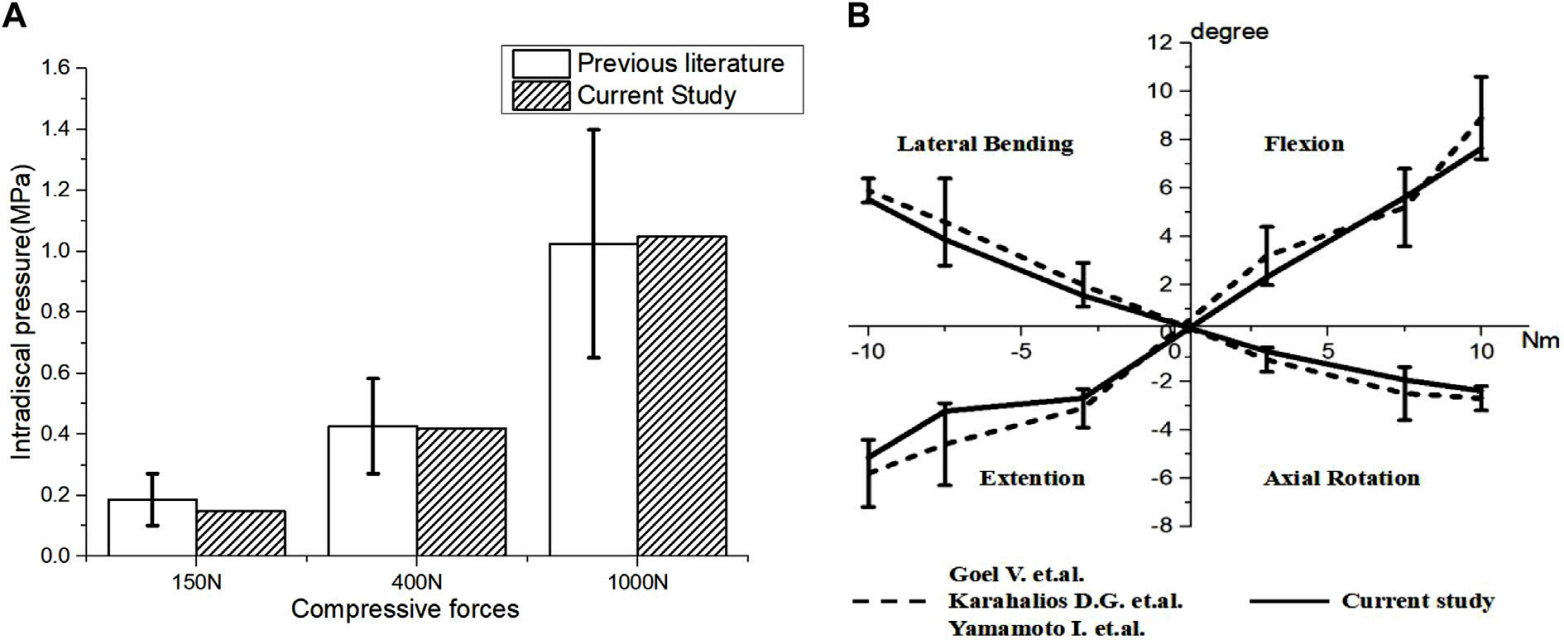
Validation of the L4–L5 spinal finite element model. **(A)** Comparison of intervertebral disc stress at the L4–L5 level and the *in vitro* experimental data from Dreischarf et al. (2014) and Brinckmann and Grootenboer, 1991, **(B)** ROM of L4–L5 lumbar spine.

The mean intervertebral disc pressure at the L4–L5 segment was 0.147, 0.419, and 1.049 MPa under compressive forces of 150, 400, and 1000 N, respectively, which were within the range of experimental data (Brinckmann and Grootenboer, 1991; Dreischarf et al., 2014). The segmental ROM under a 300 N axial load and moments of 3, 7.5, and 10 Nm was comparable to experimental results from literature (Figure 2) (Goel et al., 1985; Yamamoto et al., 1989; Karahalios et al., 2010). These results confirm the reliability of the intact FE model.

In addition, the results of the PPS model were similar to those reported in other studies. Results from Liu et al. (2020) and this study show that the ROM of the PPS model, in comparison to the intact model, was reduced by approximately 70% in flexion, extension, and lateral bending and by approximately 50% in rotation. The maximum von-Mises stress on the screws in this study was similar to findings reported by Zhang et al. (2021). This supports the validity of the PPS model in this study.

### Influence of screw inclination

**FIGURE 3:**
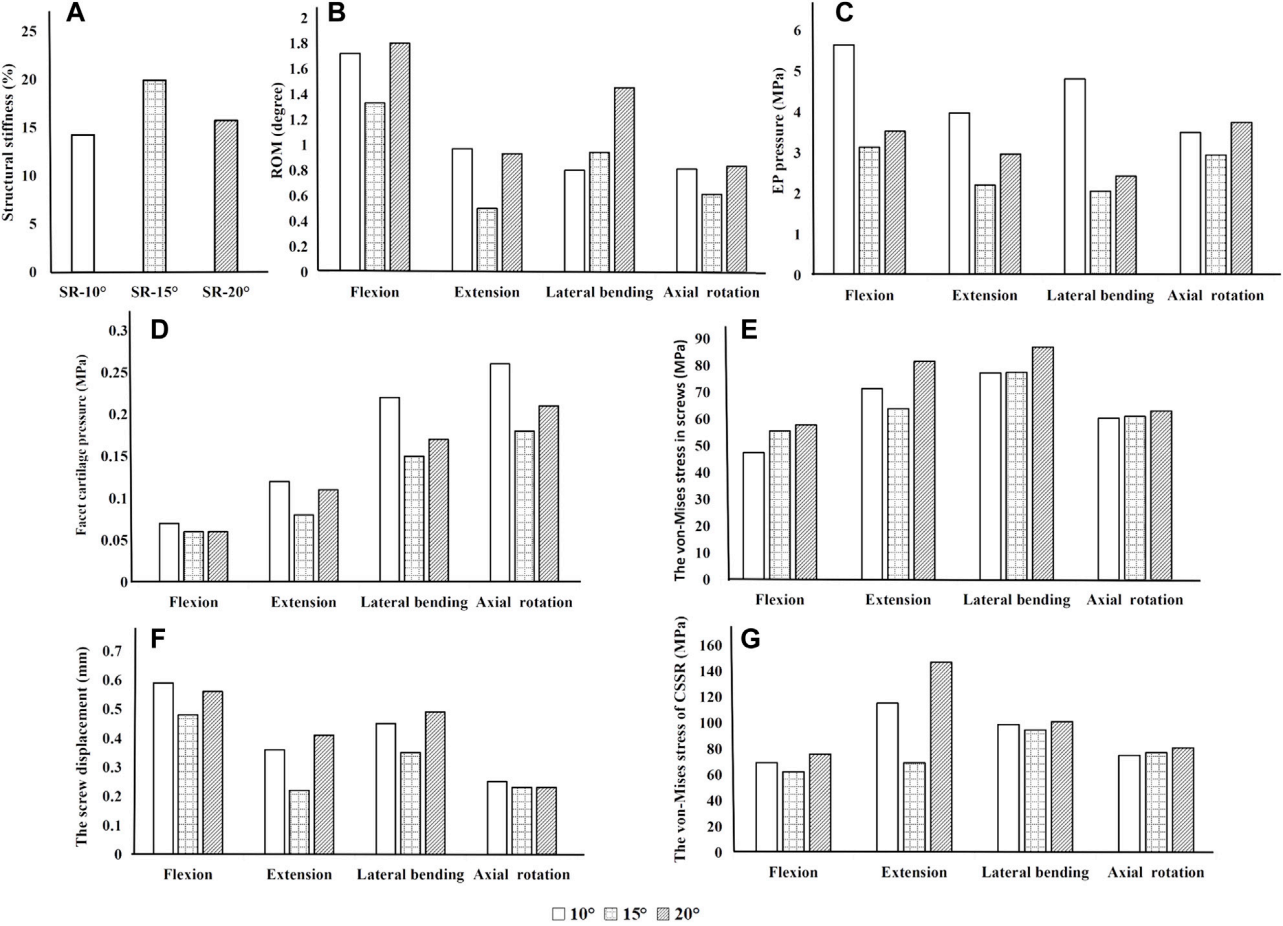
Parameters of the models for different screw inclination angles when placed under flexion, extension, lateral bending, and axial rotation. **(A)** Axial stiffness, **(B)** segmental ROM, **(C)** von-Mises stress on the endplate, **(D)** von-Mises stress on facet cartilage, **(E)** von-Mises stress on screws, **(F)** displacement of pedicle screws, and **(G)** von-Mises stress of CSSR.

As shown in Figure 3, implantation of the bilateral supports caused a significant increase in the axial compressive stiffness of the lumbar segment under a 300 N axial compressive force. Under the same load, the SR-15° model had the greatest stiffness, increasing by 19.9% over the intact spine, while the SR-20° model was 15.74% stiffer than the intact model. Correspondingly, the SR-15° model had the smallest ROM, and the SR-20° model had the largest. These results indicate that the SR-15° implantation method provided the greatest stability to the lumbar spine.

**FIGURE 4:**
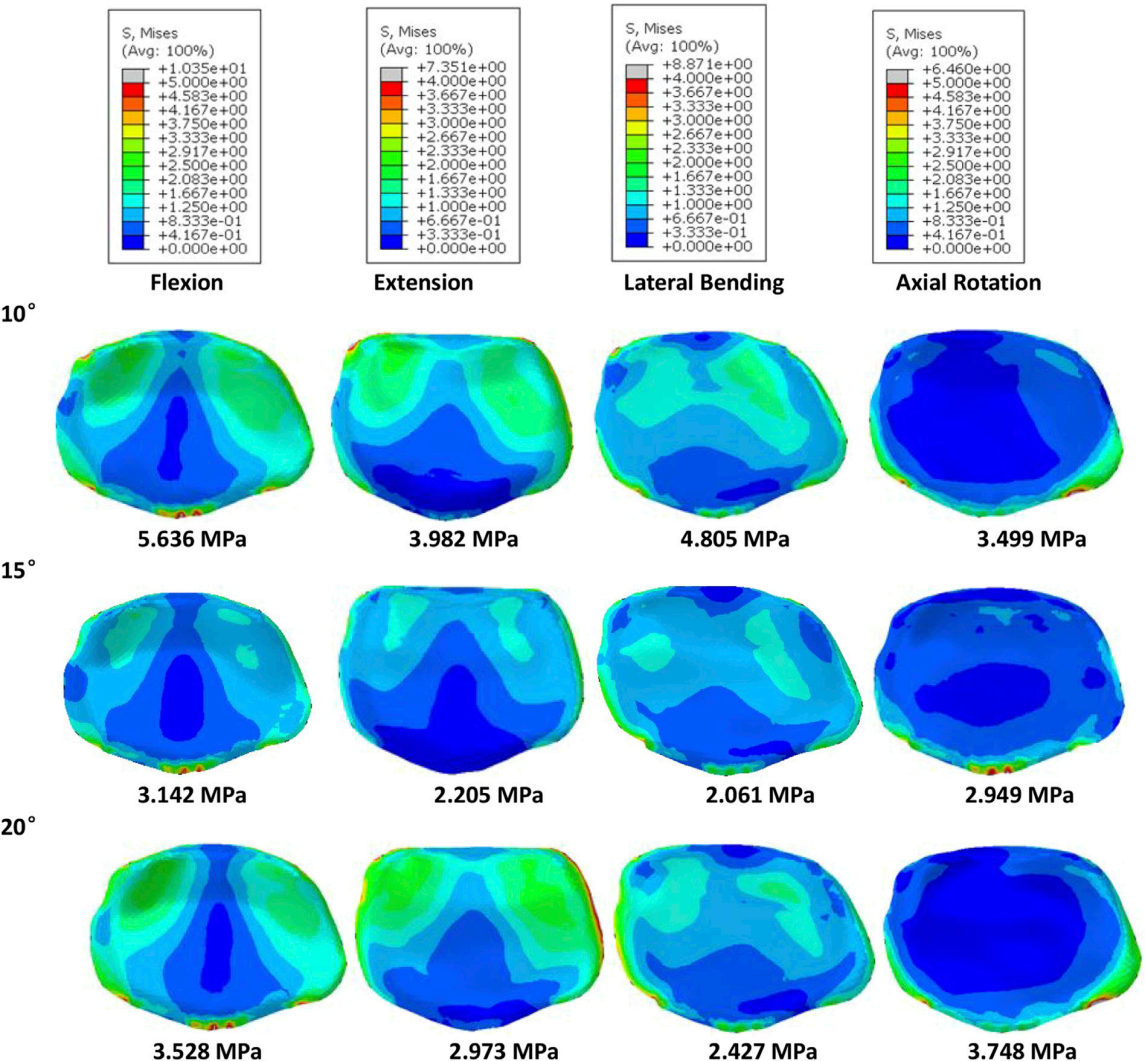
Von-Mises stress on the L4 endplate of the models for different screw inclination angles when placed under flexion, extension, lateral bending, and axial rotation, with the mean stress noted beneath each image.

**FIGURE 5:**
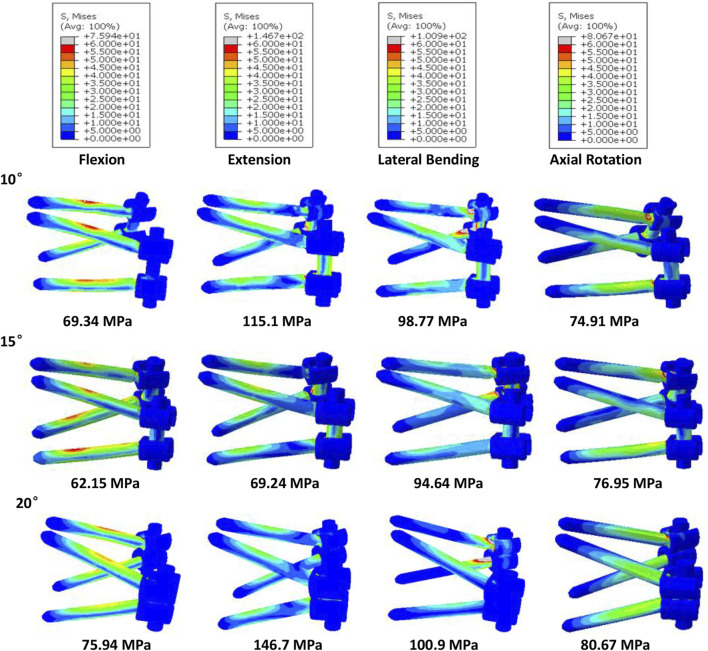
Von-Mises stress on the screws of the models for different screw inclination angles when placed under flexion, extension, lateral bending, and axial rotation, with the peak stress noted beneath each image.

Of all models and motion patterns simulated, the mean stress on the endplate was smallest in the SR-15° model, and SR-10° was the largest in flexion, extension, lateral bending, and axial rotation (Figure 4). The SR-15° model had the smallest facet cartilage stress, and SR-10° had the greatest. This demonstrated that the risk of endplate fracture and facet joint degeneration was considerably lower in SR-15° than in SR-10°. The maximum von-Mises stress on the screw increased as the angle of inclination increased, and the stress values were more evenly distributed than in other models. The SR-20° model showed the greatest stress on the screws (Figure 5). Greater screw displacement increases the risk of screw loosening. The maximum screw displacement in the three models was in flexion, and the SR-15° model had the smallest screw displacement. The SR-15° model had the lowest von-Mises stress at the CSSR increased as the angle of inclination increased, and the maximum stress for all models was located to the right of L5, shown in Figure 5.

### Influence of rod length

**FIGURE 6:**
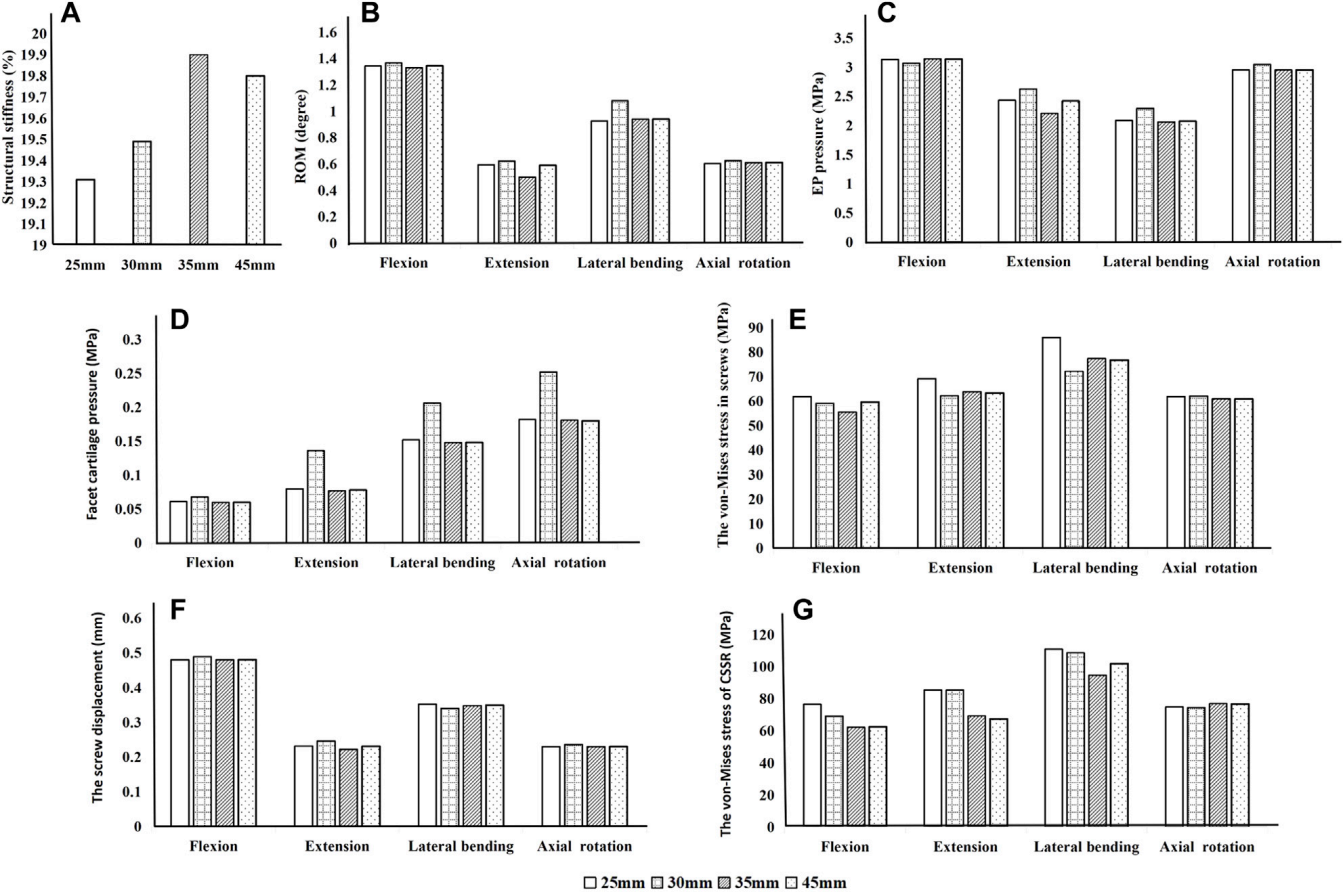
Parameters of the models for different rod lengths when placed under flexion, extension, lateral bending, and axial rotation. **(A)** Axial stiffness, **(B)** ROM, **(C)** von-Mises stress on endplates, **(D)** von-Mises stress on facet cartilage, **(E)** von-Mises stress on pedicle screws, **(F)** displacement of pedicle screws, and **(G)** von-Mises stress at the contact surface between the screw and rod (CSSR).

**FIGURE 7:**
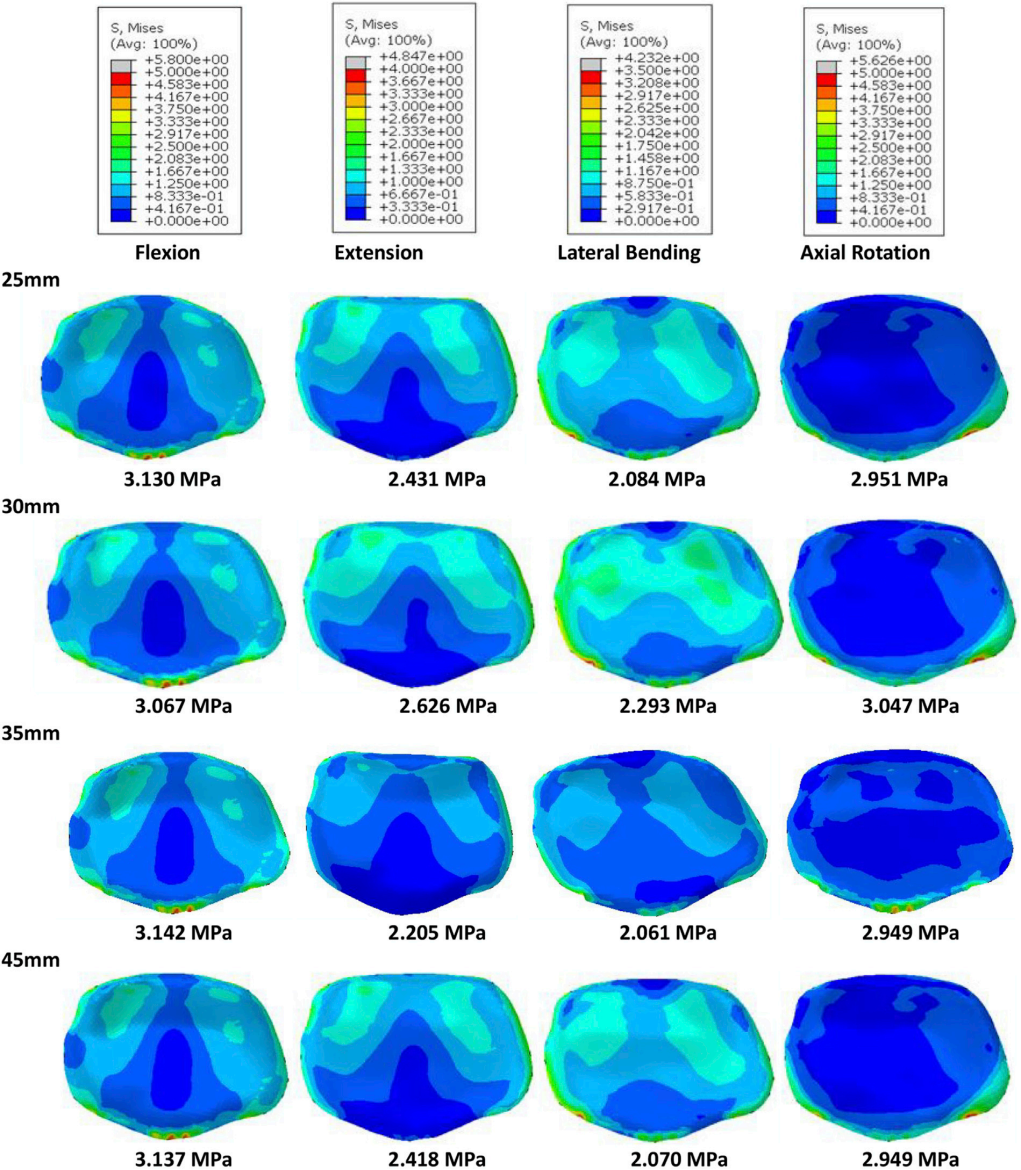
Von-Mises stress on the L5 endplate of the models for different rod lengths when placed under flexion, extension, lateral bending, and axial rotation, with the mean stress noted beneath each image.

**FIGURE 8:**
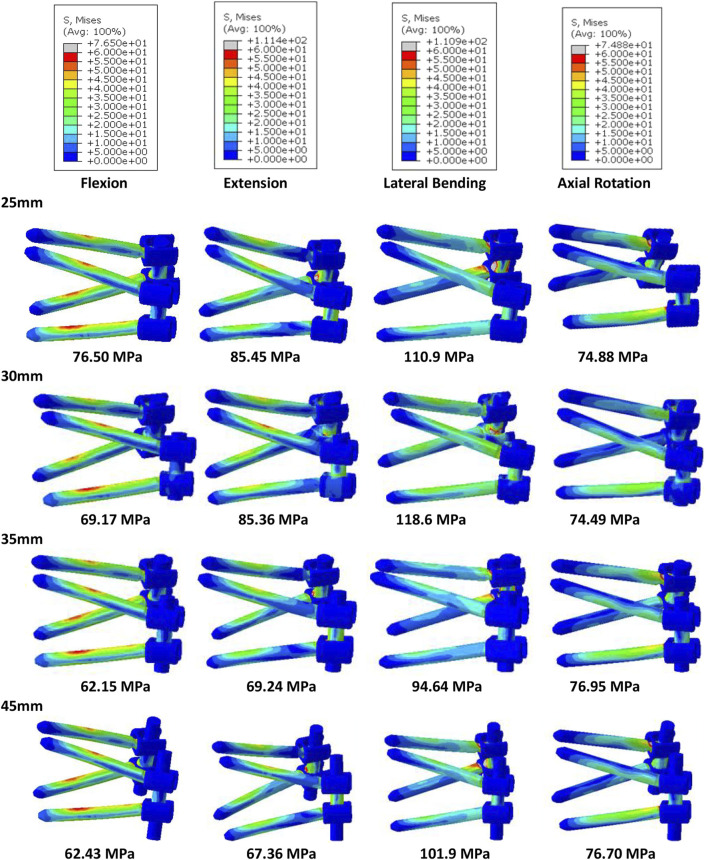
Von-Mises stress on the screws of the models for different rod lengths when placed under flexion, extension, lateral bending, and axial rotation, with the peak stress noted beneath each image.

Based on the abovementioned results, it is recommended to angle the pedicle screws at 15° to the endplate plane (SR-15°). As shown in Figure 6, under a 300 N axial load, the SR-35 mm model had the largest axial stiffness and the SR-25 mm had the smallest. There was little variation in the ROM when using different rod lengths, but SR-35 mm had the smallest ROM. These results indicate that the SR-35 mm model could offer the best restraint to the lumbar spine. The SR-30 mm model had higher stress on facet cartilage with different rod lengths. The SR-35 mm model had the lowest von-Mises stress on the endplate (Figure 7). However, the stress on the screws was smallest in extension and lateral bending for SR-30 mm, and the screw displacement was relatively large (Figure 8). The von-Mises stress at the CSSR was smallest in the SR-35 mm model and was evenly distributed.

### Feasibility of the short rod technique

**FIGURE 9:**
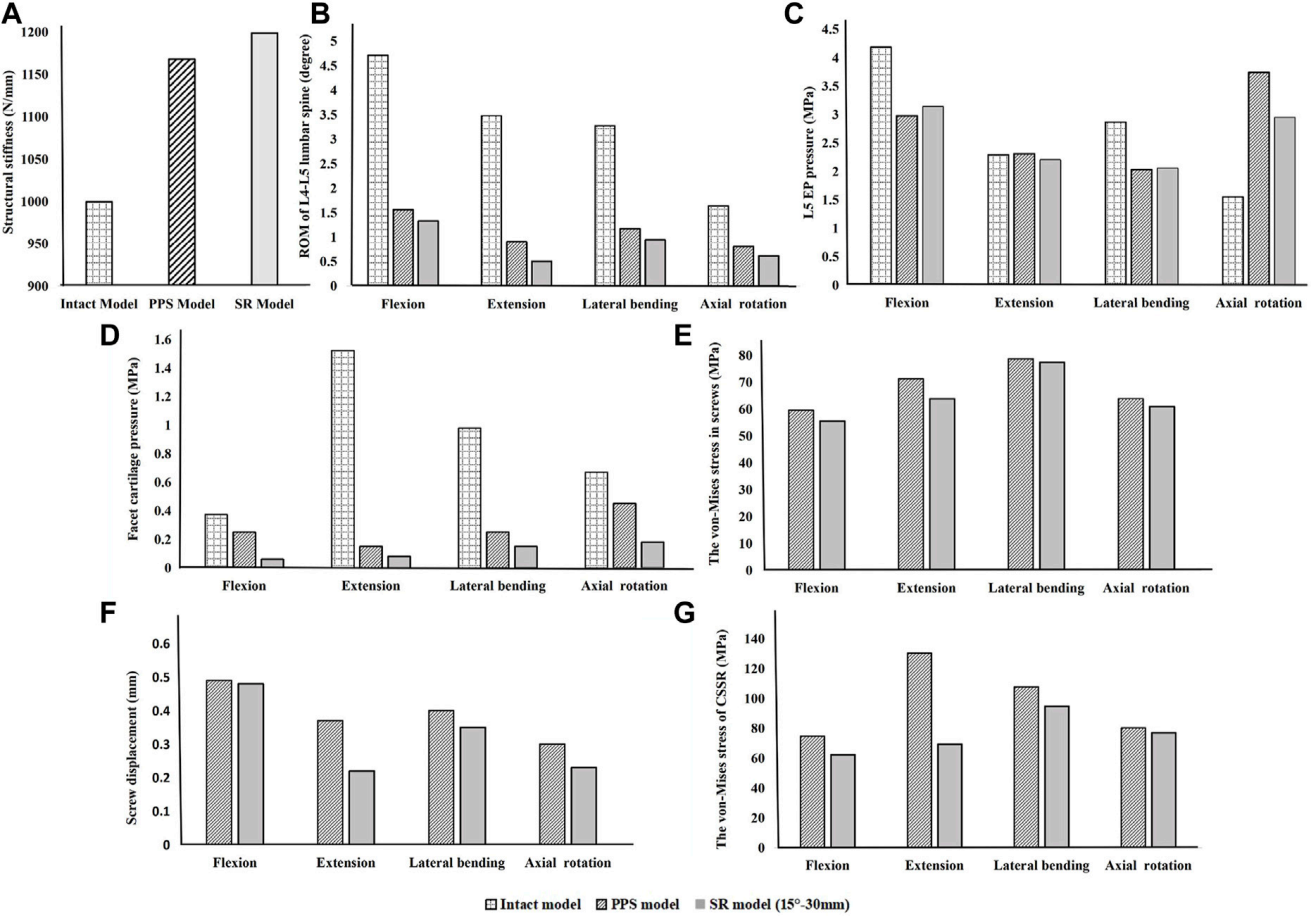
Comparison of the intact model, PPS model, and SR model. **(A)** Axial stiffness, **(B)** ROM, **(C)** endplate pressure, **(D)** von-Mises stress on facet cartilage, **(E)** von-Mises stress in screws, **(F)** displacement of pedicle screws, and **(G)** von-Mises stress at CSSR under flexion, extension, lateral bending, and axial rotation.

**FIGURE 10:**
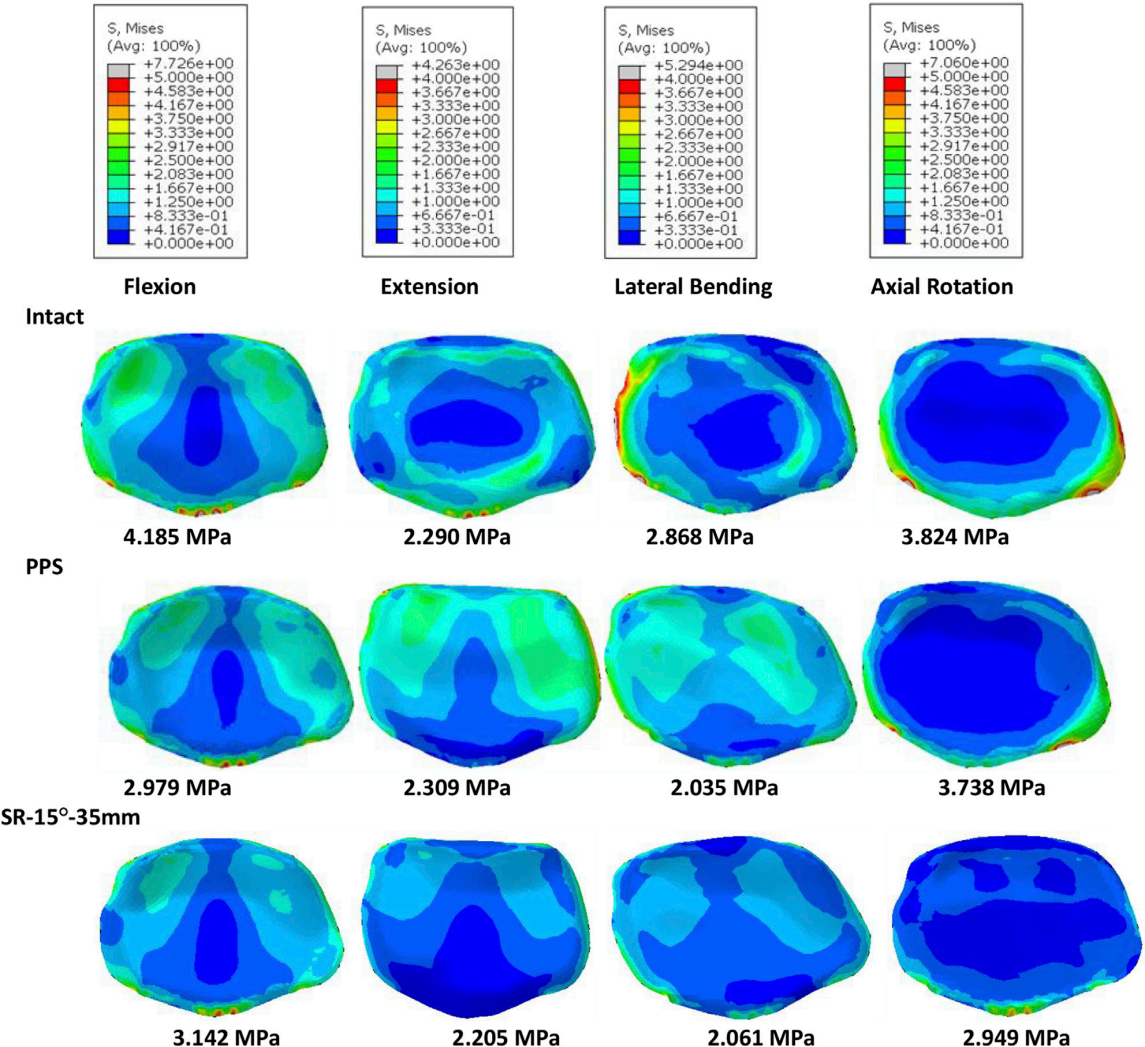
Von-Mises stress on the L4 endplate of the intact model, PPS model, and SR model, with the mean stress noted beneath each image.

Due to the excellent performance of the bilateral fixation system with 15° screw inclination and 35 mm rod, these parameters were used incorporated into the Short‐rod (SR) technique to analyze the stability of the fixation and risk of complications. As shown in Figure 9, after implantation, the axial stiffness of the models increased, while there was a reduction in the ROM, endplate stress, and facet cartilage stress. The axial stiffness of the SR model was slightly higher than that of the PPS model. The SR model had a smaller ROM and was more restricted than the PPS model. The endplate stress in the SR model was slightly lower than in the PPS model in extension and axial rotation (Figure 10). Similarly, the SR model showed lower stress on the facet cartilage than the PPS model.

**FIGURE 11:**
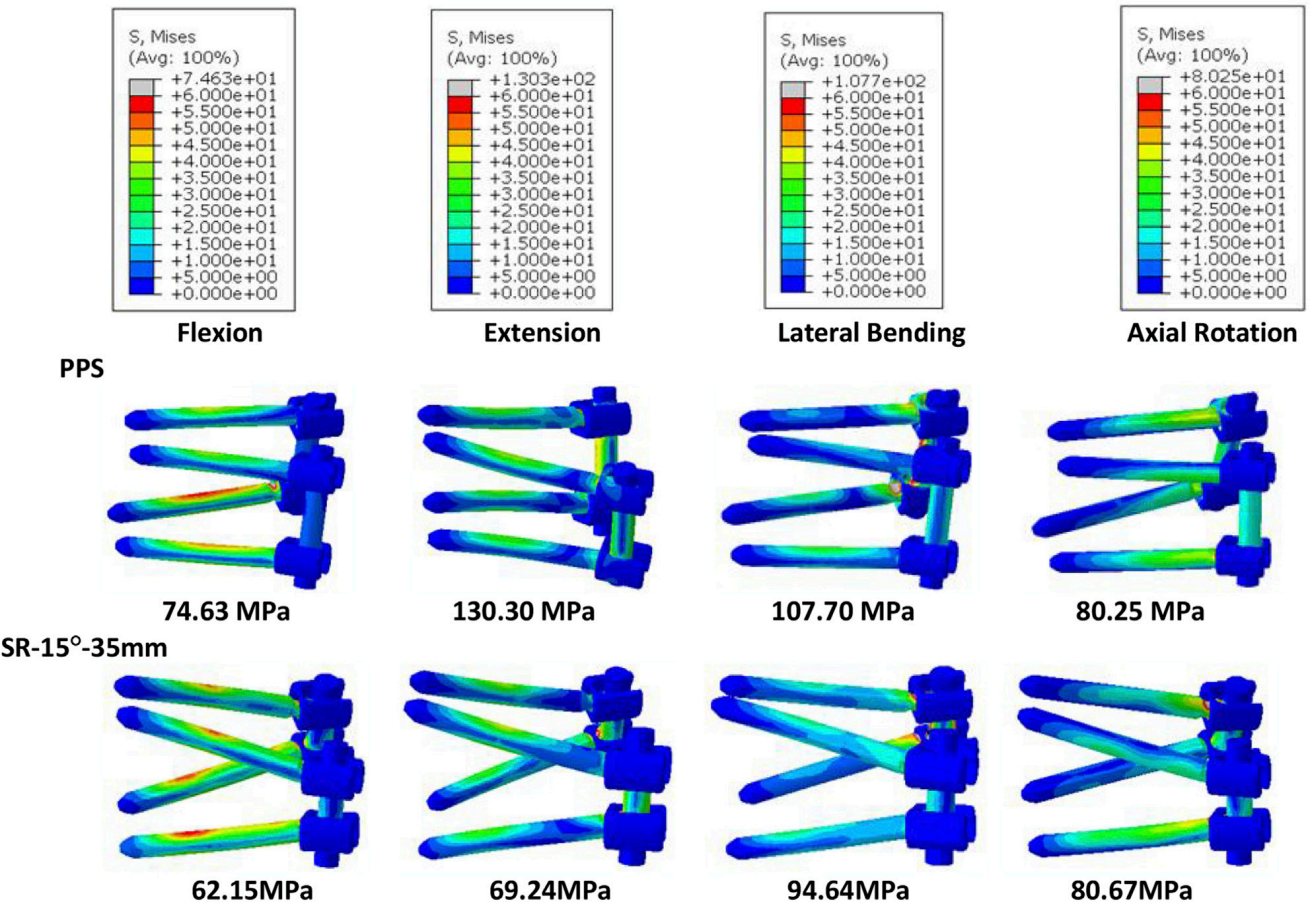
Von-Mises stress on screws of the PPS model and SR model, with the peak stress noted beneath each image.

The SR model had lower screw stress than the PPS model (Figure 11). The SR model also produced smaller screw displacement in all motions. The stress at the CSSR of the SR model was lower, and the maximum von-Mises stress occurred to the right of L5, implying a higher risk of fracture.

## Discussion

This study aimed to assess lumbar stability when using the Short‐rod (SR) technique at the L4–L5 level in a validated finite element model (FEM). The SR method allows for a smaller incision because of the angled screw insertion and shorter rod required for fixation. In this study, the setup with a 15° screw angle and a rod with 5 mm protrusion from the screw head offered the greatest lumbar stability and lowest risk of complications and thus is recommended for clinical use. The results show that the SR technique is more stable than the traditional PPS technique and carries less risk of facet joint degeneration, but this method also has a higher risk of endplate fracture in flexion and lateral bending.

The placement of screws in the vertebrae should be carefully considered when planning surgery because incorrect or unsuitable placement can have a major impact on clinical outcomes and patient mobility. Inappropriate placement may also compromise the structural stability of the lumbar spine, increasing the risk of fracture and screw loosening. This study assessed the stability of the lumbar spine and risk of complications when screws were inserted at 10° (SR-10°), 15° (SR-15°), and 20° (SR-20°) relative to the vertebral endplate. The SR-15° model has the greatest stiffness and the smallest ROM, indicating a more stable spine. Once the screw type is selected, the major factors affecting the holding power of pedicle screws are bone density and insertion angle (Varghese et al., 2017; Varghese et al., 2018). Pull-out strength predicts the initial stability of the fusion constructs post operation. Poorer bone quality with lower pull-out strength has been reported in many pieces of literature (Varghese et al., 2017). However, the effect of insertion angle is still controversial. Varghese et al. reported the pull-out force decreases with an increase in insertion in synthetic cancellous bone (Varghese et al., 2017), but (Farshad et al., 2014) reported that increasing the angle of the screws can enhance the pull-out strength with animal bone. Different materials may lead to completely different conclusions. Tilting the screws was found to increase the compressive stiffness and reduce the ROM in this study by a validated human lumbar spine model. The result seems to support Farshad’s conclusion more, perhaps because of the more similar material properties. However, the SR-15° model was significantly more stable than SR-20°. This may be due to the shorter projection length of screws in the sagittal plane of the SR-20° model, which would reduce contact with the anterior column of the vertebral body. An assessment of potential instrument-related complications showed that SR-15° had the lowest stress on the vertebral endplate and facet cartilage, which is expected to reduce the risk of fracture and degeneration. A high number of cases of lower back pain in the general population are associated with endplate fracture in daily life (DieeN et al., 1999), while the SR-15° approach may reduce the incidence of back pain due to the lower risk of endplate fracture, but it still needs to be further assessed through a clinical study. High stress concentrations can lead to breakage of the fixation system (Newcomb et al., 2017), so the high stress observed on the CSSR to the right of L5 is of particular concern. As the screw angle increases, so does the risk of screw breakage. SR-15° was observed with moderate stress levels on the screws, more uniform stress distribution, and smaller screw displacement than both SR-10° and SR-20°, which can effectively reduce the risk of complications related to screw fixation. Krag et al. (1988) found that screw displacements over 0.7 mm could lead to trabecular bone damage, and all models in this study recorded a screw displacement less than 0.59 mm with little risk of screw loosening. Although we did not perform pull-out experiments, we predict that SR-15° has better pull-out strength based on the higher lumbar stiffness and less screw displacement. In addition, when placing the screw, this study found that a 20° inclination may cause the tail of the screw to collide and increase the risk of intraoperative penetrating the vertebral endplate or damaging the pedicle because the lumbar spine is physiologically lordotic (Yang et al., 2019), which greatly increases the difficulty of the operation.

This study also compared the biomechanical characteristics of different rod lengths. With the SR method, the screw on the ipsilateral side is at an acute angle. Using a uniaxial screw will result in greater screw reduction force and more axial slip and is more prone to axial slip than uniplanar and polyaxial screws (Serhan et al., 2010). Therefore, polyaxial screws were used in this study, which also allowed the screw head to be angled to accommodate the rod to improve seating and reduce axial slip. To assess the effect of rod length on stability, we compared stiffness and ROM across the four groups. The results show that SR-25 mm had lower stiffness than the other three groups, indicating a poor overall resistance to deformation. This may be due to the shorter rods that do not extend beyond the distal screw heads, causing inadequate engagement with the screw head (Serhan et al., 2010). In contrast, the longer rods that extended past the screw head could provide a buffer for rod slippage. The SR-35 mm and SR-45 mm constructs had a lower ROM. This study also assessed the relationship between rod length and risk of complications. The results showed that SR-35 mm had the smallest screw displacement and screw stress, while SR-25 mm had high screw stress, suggesting that the longer rod length may decrease the force on the screw. However, SR-35 mm and SR-45 mm had little difference on the stress on the endplates, facet cartilage, or CSSR, while SR-45 mm requires a larger incision for implantation. Although there was no significant difference in results between groups with different rod lengths, this study recommends using the SR-35 mm setup based on the numerical superiority of the results.

Based on the assessment of biomechanical characteristics of the lumbar spine when using different screw inclinations and rod lengths, we recommend a 15° screw inclination and a rod length of 35 mm for the SR technique.

This study evaluated the stability of the SR technique by comparing the axial stiffness and ROM against the PPS technique. The results showed that the SR technique has greater stiffness and a smaller ROM, which can provide better stability to the vertebrae. The pedicle is the strongest part of the vertebra due to the surrounding hard cortical bone, and the inclination of the screws increases the contact area with the cortical bone (Xi et al., 2021), which may enhance the biomechanical stability of the SR technique. İnceoğlu et al. (2011) also found that increasing the contact area between the screw and cortical bone can increase the screw pullout resistance. Additionally, the SR technique requires a smaller incision than the PPS method, resulting in less disruption of the lumbar musculature. Since the lumbar musculature plays an important role in stability (Panjabi, 2003), it is possible that the SR technique will result in a more stable spine post-operatively and bring shorter fusion times.

Securing a segment of the lumbar spine will result in a stress differential between fixed and non-fixed segments, which is accepted as the main factor in the development of adjacent segment degeneration (ASD) (Hikata et al., 2014; Park et al., 2018). The excellent stability and stiffness of SR constructs can reduce the risk of ASD and improve the long-term clinical outcome. In addition, the entry point of screws with the PPS method is close to the facet joint of the adjacent segment, and there is a risk of damage to the articular facet, which can lead to further degeneration of ASD (Wang et al., 2022).

While playing an important role in the physiological function of the lumbar spine, facet joints also carry 6%–30% of the axial compressive load (Yin et al., 2020). This loading may also accelerate the degeneration of the facet joint, especially in people over 60 years of age (Yin et al., 2020). Such degeneration usually begins with changes in the articular cartilage and eventually leads to destruction of the entire joint and tissues. This study found that the SR method resulted in less facet cartilage stress than PPS in the surgery segment, which could reduce degeneration of the facet joint.

The fracture of the endplate is one of the common complications after lumbar fusion surgery, reported with a rate of 2.2%–14.6% (Inoue et al., 2021), and osteoporotic spines and older females are recognized as being at heightened risk (Nakahashi et al., 2019). This study found that stress on the endplate was significantly reduced and the stress was more evenly distributed under the action of the screw than in the intact model. Lower endplate stress and fewer stress concentrations would reduce the incidence of endplate fracture. The SR technique allows for lower endplate stress in extension and axial rotation but higher in flexion and lateral bending, meaning that the SR technique should be used cautiously in people prone to fracture, such as in osteoporotic patients.

Screw loosening and breakage are also common complications associated with pedicle screws, occurring in 0.8%–27% of cases, and may exceed 50% in patients with osteoporosis (Xi et al., 2021). Newcomb et al. (2017) found that using a larger insertion angle for pedicle screws may reduce the risk of screw loosening and breakage. Our study found that stresses were concentrated on the tail of the screw in the SR model, which may lead to fatigue failure around this region. However, the maximum von-Mises stress on the screws in the SR model was smaller than in the PPS model, meaning it was at a lower risk of screw fracture. The results of this study showed lower CSSR stresses with the SR method, which may also decrease the risk of screw breakage. Fragmentation of the CSSR region has been reported in the literature. A screw-rod system like the “crane” force structure determines the stress concentration at the center of rotation, where the screw and rod come into contact (Song et al., 2021). In addition to the stress on the pedicle screw, the insertion method and stability of the screw fixation system can also have a considerable effect on the healing capacity of the injured vertebra. Repeated displacement not only limits the growth of new trabecular bone but also causes resorption of grafted bone (Zhou et al., 2020). This study found that the SR technique suffered lower screw displacement than the PPS technique, suggesting this technique may carry a lower risk of screw loosening, although the difference between the two groups was not significant. The maximum screw displacement in the SR model was 0.48 mm, which was significantly less than the 0.7 mm reported by Krag et al. (1988), which could lead to trabecular bone damage and screw loosening. Future studies may also consider including longer screws for assessment, which have been reported to better constrain the fixation system (Cunningham et al., 2010).

The SR technique can provide better initial stability and lower the risk of facet joint degeneration. Also, because of the minimally invasive approach, SR can reduce iatrogenic complications caused by extensive iatrogenic paraspinal muscle injury. However, long-term screw-related complications cannot be ignored and may be improved by removing the internal fixation after fusion Tsuang et al., (2019).

There are some limitations to this study. First, the lumbar models did not consider musculature, which may affect the stability and flexibility of the spine. Second, this study did not consider the effect of rod curvature. However, since the SR technique uses a shorter rod length, the amount of bending would be negligible. Third, this study did not consider the effect of bone mineral density on stress pattern behavior. Methods to discuss the effects of internal fixation using non-osteoporotic models are widely used. In order to specifically discuss the effect of screw inclination angle and rod length on fixation, the fixation effect with different bone mineral densities will be discussed in future studies. Fourth, this study only considered screw angle and rod length and did not consider parameters such as screw length, diameter, and thread that are equally important for the stability of the fixation system. This study was to determine how the SR technique operates and evaluate the biomechanics of the SR technique. The geometric parameters of the screw can be considered in future studies to optimize the SR technique. Finally, the FEM did not directly assess the impact of SR technology on the adjacent vertebral segment, but instead used the axial stiffness of the fixed vertebrae to predict the risk of adjacent vertebral disease.

## Conclusion

This study used finite element analysis to evaluate the effects of screw inclination angle and rod length with the SR technique on the lumbar spine. The biomechanical function of the lumbar spine was compared among the intact model, PPS model, and SR model. Compared to the PPS technique, the SR technique with a 15° screw inclination to the endplates in the sagittal plane and 35 mm rod length was more stable, required a smaller incision, and presented a lower risk of facet joint degeneration, screw breakage, and screw loosening. However, the SR technique is also at a slightly higher risk of endplate fracture than the PPS technique. The results of this study support that the use of the SR technique can offer good lumbar stability with a low risk of instrument-related complication.

## Data Availability

The raw data supporting the conclusion of this article will be made available by the authors, without undue reservation.
